# COVID-19: a gray swan’s impact on the adoption of novel medical technologies

**DOI:** 10.1057/s41599-022-01247-9

**Published:** 2022-07-08

**Authors:** Denise R. Dunlap, Roberto S. Santos, Craig M. Lilly, Sean Teebagy, Nathaniel S. Hafer, Bryan O. Buchholz, David D. McManus

**Affiliations:** 1 University of Massachusetts Lowell, Lowell, MA USA; 2 Merrimack College, North Andover, MA USA; 3 University of Massachusetts Chan Medical School, Worcester, MA USA

**Keywords:** Health humanities, Complex networks

## Abstract

The COVID-19 pandemic offers a unique context and opportunity to investigate changes in healthcare professional perceptions towards the adoption of novel medical technologies, such as point-of-care technologies (POCTs). POCTs are a nascent technology that has experienced rapid growth as a result of COVID-19 due to their ability to increase healthcare accessibility via near-patient delivery, including at-home. We surveyed healthcare professionals before and during COVID-19 to explore whether the pandemic altered their perceptions about the usefulness of POCTs. Our network analysis method provided a structure for understanding this changing phenomenon. We uncovered that POCTs are not only useful for diagnosing COVID-19, but healthcare professionals also perceive them as increasingly important for diagnosing other diseases, such as cardiovascular, endocrine, respiratory, and metabolic diseases. Healthcare professionals also viewed POCTs as facilitating the humanization of epidemiology by improving disease management/monitoring and strengthening the clinician-patient relationship. As the accuracy and integration of these technologies into mainstream healthcare delivery improves, hurdles to their adoption dissipate, thereby encouraging healthcare professionals to rely upon them more frequently to diagnose, manage, and monitor diseases. The technological advances made in POCTs during COVID-19, combined with shifting positive perceptions of their utility by healthcare professionals, may better prepare us for the next pandemic.

## Introduction

“…no matter how many instances of white swans we may have observed, this *does not justify the conclusion that all swans are white.” — Karl Popper*

Given the prevalence of white swans, the sighting of a black swan is considered by many to be an unpredictable event. Therefore, no matter how many white swans an individual sees, they rarely expect the next swan they see to be black (e.g., Murphy et al., 2020). Global pandemics (e.g., COVID-19) are often considered and treated as black swans (e.g., Atkinson-Clement and Pigalle, 2021). However, prior close calls with other pathological viruses including SARS, MERS, and Ebola that are transmitted among and afflict human beings, indicated that another pandemic outbreak was bound to occur again. In an era of globalization, it should not have come as a surprise that what began as a localized health issue would quickly spread around the world (Collins, 2020). Similar to a black swan, the COVID-19 pandemic was difficult to predict and continues to have an unknown long-term impact on all aspects of society. However, since it was inevitable given prior history (e.g., Spanish Flu), it can be considered as a “gray swan” event (Collins, 2020; Taleb, 2010). Gray swan events such as these (Phan and Wood, 2020; Taleb, 2010) shift attitudes and behaviors about how societies administer and provide access to healthcare as well as offer unique opportunities to stimulate innovation (Harris et al., 2020).

The magnitude of COVID-19 and its consequences have been far-reaching and revealed limitations in traditional healthcare delivery systems. One key weakness was the dependence on laboratory-based diagnostic technologies. To avoid potential exposure to COVID-19, an increasing number of patients forwent routine care visits in which traditional laboratory-based diagnostic and surveillance testing was conducted (Dinmohamed et al., 2020; Kaufman et al., 2020), resulting in a healthcare delivery gap (Dunlap et al., 2021).

In response, governments funded programs, such as the National Institutes of Health’s (NIH’s) Rapid Acceleration of Diagnostics (RADx) initiative, to enable companies to rapidly develop and deploy novel medical technologies to address patient care through decentralized, diagnostic point-of-care tests (POCTs) (Schachter et al., 2021). The central benefit of POCTs is that they can be performed by individuals without specialized training at sites other than traditional laboratories (e.g., in the clinic, ER, hospital, or home setting), potentially being performed without a prescription and can provide timely results directly to the user.

In recent years, POCTs have been used more extensively to expedite the diagnosis of pregnancy, test blood glucose levels, monitor hypertension, and provide remote care for the elderly (Dang et al., 2009; Ding et al., 2019). In the case of diabetes, for example, timely blood glucose testing is essential for patients to be able to actively monitor their condition in real-time to avoid macrovascular (e.g., the large blood vessels of the brain, heart, and legs) and microvascular (e.g., the small blood vessels of the kidneys, eyes, and feet) complications. Timely and accurate POCTs reduce variation in testing outcomes, which offers patients the ability to better manage their condition and enhance their quality of life. Thus, the development and adoption of POCTs to diagnose these as well as other infectious diseases, including sexually transmitted infections (STIs) is critically important. Yet, according to the literature, and as COVID-19 has shown the world, the technological trajectory and potential usefulness of POCTs is still in its infancy (Gaydos et al., 2021; Toskin et al., 2017).

Driven by advances in novel medical technologies, an unintended consequence of the pandemic was the change in perceptions about the usefulness of POCTs among healthcare professionals and their patients (e.g., Schachter et al., 2021) not only in terms of their utility for combatting the pandemic, but also for other diseases. Yet, there remains of dearth of POCT research in the literature. There are a relatively few studies that have explored the factors influencing POCT adoption by healthcare professionals (see Dunlap et al. (2021) and Teebagy et al. (2022) for recent studies). Further, while there are a number of papers that look at COVID-19 now, few studies offer the opportunity to analyze and derive insights about new technologies before and after COVID-19. Thus, many questions remain unanswered as to how gray swan events, such as the COVID-19 pandemic, may impact healthcare professionals’ perceptions about the adoption of novel POCT technologies to diagnose and monitor not only infectious diseases, but how this may also spill over to other disease categories as well.

To understand this shift, we leverage a unique asset—a survey administered to healthcare professionals about their perceptions of POCTs. An identical survey was administered both before and one-year into the COVID-19 pandemic as part of the National Heart, Lung, and Blood Institute (NHLBI)-funded Center for Advancing Point-of-Care Technologies (CAPCaT) annual solicitation to healthcare professionals for feedback on its call for research proposals. Our study explores how healthcare professionals’ perceptions of POCTs changed as they pertain to critical technological characteristics (e.g., accuracy, speed, cost), as well as their ability to diagnose, manage, and monitor diseases. Our survey of healthcare professionals found that they responded favorably to this nascent technology and saw greater utility in its applicability towards cardiovascular, endocrine, respiratory, metabolic, and infectious diseases.

The implications of this study reveal that healthcare professionals are becoming increasingly open to the idea of shifting from provider-initiated patient care to empowering patients to become active participants and advocates of their own healthcare via the adoption of novel POCT technologies. In this regard, POCTs can help further the humanization of epidemiology by facilitating greater dialog between clinicians and patients, thereby strengthening this important relationship.

## Methods

### Study population and recruitment

To collect the data for this study, we employed a survey approach. Through an iterative process with an expert panel of healthcare professionals, we created several survey questions to assess healthcare professional perceptions of the conditions for which POCTs could be helpful. In particular, we asked respondents to (1) “Name up to five conditions for which a POCT could help you make a DIAGNOSIS of a disease” and (2) “Name up to five conditions for which a POCT could help you MONITOR or MANAGE disease.” These were structured as open text fields for respondents to record their responses. We also asked respondents (3) “Which characteristic of a point of care technology is most important when incorporating it into your regular practice?” This was structured as a radio button from which respondents could select one of 12 different options: availability, ease of use, accuracy, sample type, sample collection, does not disrupt workflow, cost, device footprint, reimbursement for testing, information systems connectivity, CLIA-waived status, and ruggedness. We also collected data on respondent demographics, including gender, race, profession, specialty, years in practice, and practice environment. The University of Massachusetts Chan Medical School’s (UMass Chan) Institutional Review Board (IRB) deemed this study to be exempt from review in July 2019 (docket H00018195).

Survey accuracy was validated using a focus group of 10 healthcare providers, clinicians, and UMass Chan faculty and their feedback was used to identify survey items that accurately captured their views. To circumvent the possibility of common method variance bias influencing our results (Podsakoff et al., 2003), we divided the survey into two waves that were administered approximately six-months apart. Respondents accessed the survey via an email link to a secure instance of the REDCap data management platform, which was hosted on an encrypted UMass Chan server that could only be accessed by authorized individuals.

The first survey was distributed from October 8, 2019 to March 25, 2020 (we will refer to this survey hereafter as T1) and the second, identical survey, was distributed from October 29, 2020 to November 30, 2020 (we will refer to this survey hereafter as T2). This timing means that nearly all T1 responses occurred before widespread circulation of COVID-19 (the disease caused by the SARS-CoV-2 virus) in the United States, while T2 was collected in the early stages of the third surge of COVID-19 cases. The survey was specifically targeted towards healthcare professionals, researchers, and medical device developers and was sent to 16 internal and external email directories (including the University of Massachusetts Center for Clinical and Translational Science, Massachusetts Medical Device Development Center (M2D2), Consortia for Improving Medicine with Innovation & Technology (CIMIT), Center for Advancing Point of Care Technologies (CAPCaT), National Heart, Lung, and Blood Institute (NHLBI), Small Business Innovation Research (SBIR), NIH Center for Accelerated Innovations (NCAI), Research Evaluation and Commercialization Hubs (REACH), and National Center for Complementary and Integrative Health (NCCIH)), which included over 15,000 individuals, although the precise number is unknown. Additionally, emails were sent to 172 individuals identified through targeted searches using the keywords ‘point of care’ on the National Institutes of Health (NIH) RePORTER^1^ website and ‘point of care,’ ‘point of care heart,’ point of care lung,’ ‘point of care blood,’ and ‘point of care sleep’ on the UMass Chan Medical Profiles^2^ and Direct2Experts^3^ websites. Additional outreach efforts were conducted to recruit survey participants in November 2019 at the Healthcare Innovations and Point-of-Care Technologies Conference, held in Bethesda, Maryland, and via a LinkedIn post. We sent out a reminder email to survey non-respondents 2 weeks after the initial invitation was distributed. We also conducted interviews with UMass Chan medical researchers between March and September 2021. Our exclusion criterion omitted survey respondents who did not self-identify as a healthcare professional.

### Sample and coding

We collected a total of 149 valid questionnaires from the T1 survey (~1% response rate) and a total of 286 valid questionnaires from the T2 survey (~2% response rate), which resulted in a total sample of 435 valid questionnaires.^4^ The majority of survey respondents were from the United States (T1 = 99.3% from U.S. while T2 = 98.6% from U.S.). Non-U.S. respondents were located primarily in India, Greece, Iran, and the United Kingdom. The survey respondents’ demographic information is presented in Table 1 for both survey samples.

**Table 1:** Demographic information of respondents.

Characteristics	T1 *N* = 149 Qty. (%)		T2 *N* = 286 Qty. (%)	
Gender				
Female	63	(42.3)	120	(42.0)
Male	77	(51.7)	154	(53.9)
No response	9	(6.0)	12	(4.2)
Race				
White	103	(80.5)	192	(75.9)
Black	2	(1.6)	9	(3.6)
Asian	22	(17.2)	50	(19.8)
Native American	0	(0.00)	1	(0.4)
Other	1	(0.8)	1	(0.4)
Specialty				
Cardiology	25	(16.5)	48	(16.8)
Family or internal medicine	23	(15.1)	39	(13.6)
Pulmonology	11	(7.2)	71	(24.8)
Hematology	4	(2.6)	4	(1.4)
Emergency medicine	22	(14.5)	19	(6.6)
Sleep medicine	8	(5.3)	12	(4.2)
Other	59	(38.8)	111	(32.5)
Profession				
Medical doctor	83	(55.7)	163	(57.0)
Doctor of osteopathy	5	(3.4)	8	(2.8)
Nurse practitioner	6	(4.0)	12	(4.2)
Advanced practice nurse	1	(0.7)	4	(1.4)
Physician’s assistant	0	(0.0)	4	(1.4)
Registered nurse	21	(14.1)	37	(12.9)
Other	26	(17.5)	52	(18.2)
No response	7	(4.7)	6	(2.1)
Practice environment				
In-home	3	(2.0)	8	(2.8)
Ambulatory clinic	28	(18.8)	72	(25.2)
ER	16	(10.7)	16	(5.6)
In-hospital	74	(49.7)	151	(52.8)
Other	20	(13.4)	29	(10.1)
No response	8	(5.4)	10	(3.5)
Years of practicing				
0–5 years	47	(31.5)	65	(22.7)
6–10 years	16	(10.7)	51	(17.8)
11–15 years	18	(12.1)	37	(12.9)
16–20 years	21	(14.1)	36	(12.6)
Over 20 years	37	(24.8)	88	(30.8)
No response	10	(6.7)	9	(3.2)

Across both surveys, the majority of respondents were male (T1 = 51.7%, T2 = 53.9%), identified as white (T1 = 80.5%, T2 = 75.9%), were medical doctors (T1 = 55.7%, T2 = 57.0%), and practiced in-hospital (T1 = 49.7%, T2 = 52.8%). However, there were also some differences between the respondents of the two survey waves. In particular, T1 saw more responses from emergency medicine specialists (T1 = 14.5 vs. T2 = 6.6%) and individuals with 0-5 years of experience (T1 = 31.5 vs. T2 = 22.7%) while T2 saw more responses from pulmonology specialists (T2 = 24.8 vs. T1 = 7.2%) and individuals with over 20 years of experience (T2 = 30.8 vs. T1 = 24.8%).

The survey responses included medical conditions such as diabetes, Hepatitis C, atrial fibrillation, urinary tract infections (UTI), and pregnancy among many others. We invited two independent medical researchers to participate in the coding of the medical condition data. First, the two researchers independently categorized each medical condition into one of 23 therapeutic categories (comprising a total of 720 medical conditions) consistent with the therapeutic categories used by Biomedtracker. We then compared their categorizations of the medical conditions.^5^ In terms of inter-rater reliability, there was a greater than 98% agreement in the categorizations made by the two researchers. The occasional difference in categorization was adjudicated by a third independent medical researcher with over 20-years of experience.

### Analytical approach

Given the nature of the data collected and our research design, network analysis was used to explore not only the relationships between survey participant responses both before COVID-19 (i.e., T1) and during COVID-19 (i.e., T2), but also the structures that emerge as a result of recurring relationships. Location in the center of the network (i.e., centrality) suggests that a condition is of greater importance, whereas being located on the periphery of the network typically denotes that a condition was deemed of lower importance. In our network analysis, the size of nodes (i.e., the circles) represent the frequency with which the condition was reported by respondents. The thickness of the connections (or “edge”) represents the strength of the relationship between different conditions. The thicker the connection (or “edge”), the more frequently a first condition was reported in conjunction with a second condition. The arrowhead shows the direction of the relationship. For example, an arrowhead that points from condition A to condition B means that respondents who identified condition A as their primary concern also identified condition B as another condition of concern. A line with arrows on both ends indicates a mutual relationship between conditions. The length of the arrow has no meaning in the display of the data. We present our network analysis results using a Fruchterman-Reingold layout (Fruchterman and Reingold, 1991), which uses a force-directed algorithm to produce a layout that is easy to visualize.

## Results

**Fig. 1:**
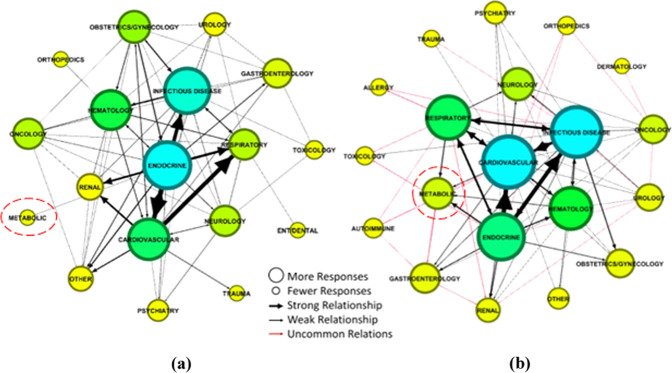
Network analysis of healthcare professional perceptions of the medical conditions for which POCTs would be most helpful to diagnosis a disease. **a** Before COVID-19, healthcare professionals identified endocrinology as the condition for which POCTs was most helpful for making a medical diagnosis. **b** During COVID-19, healthcare professionals were focused more on using POCTs for diagnosing infectious diseases.

Figure 1 presents the network analysis of healthcare professional perceptions of the medical conditions for which POCTs would be most helpful to diagnosis a disease. Figure 1, panel a illustrates the network analysis of the T1 survey (533 reported conditions). Prior to COVID-19 being designated as a pandemic, healthcare professionals identified 18 medical condition categories for which POCTs could be used to diagnose a disease. Endocrine disorders, including diabetes mellitus, featured prominently in the center of the network, followed by infectious disease, cardiovascular, and hematology conditions; suggesting that healthcare professionals considered these conditions to be the most important for which POCTs could be beneficial for diagnosing a disease pre-pandemic. The strongest relationships were observed between endocrine ↔ cardiovascular, endocrine → infectious disease, and cardiovascular → respiratory.

Figure 1, panel b illustrates the network analysis of the T2 survey (1118 reported conditions). Following COVID-19’s designation as a pandemic, healthcare professionals identified 20 medical condition categories for which POCTs could be used to diagnose a disease. Infectious disease and cardiovascular-related medical conditions featured prominently in the center of the network, followed by endocrine, respiratory, and hematology conditions; suggesting that healthcare professionals considered these conditions best suited for POCTs to improve their ability to diagnose a disease in their practice. The strongest relationships were observed between endocrine ↔ cardiovascular, endocrine → infectious disease, and cardiovascular → infectious disease. There is a consequential difference between clinician perspectives towards using POCT devices for medical diagnosis pre- and during COVID-19. The red connections (or “edges”) indicate relationships between conditions during COVID-19 that are not common with those pre-COVID-19.

**Fig. 2:**
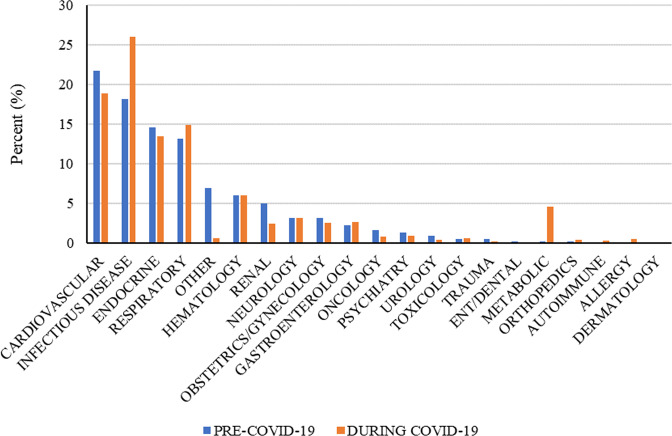
During COVID-19, the infectious disease and metabolic categories experienced the greatest percentage increase in healthcare professional attitudes towards using POCTs to diagnose diseases.

Referring to Fig. 2, following COVID-19’s designation as a pandemic, there was a 7.8% increase in the infectious disease category (*t* = −3.519, *p* < 0.01) and a 4.4% increase in the metabolic category (*t* = −4.788, *p* < 0.01). Thus, there was a substantial increase in healthcare professional attitudes towards the use of POCTs to diagnose medical conditions in these two categories during the pandemic.

**Fig. 3:**
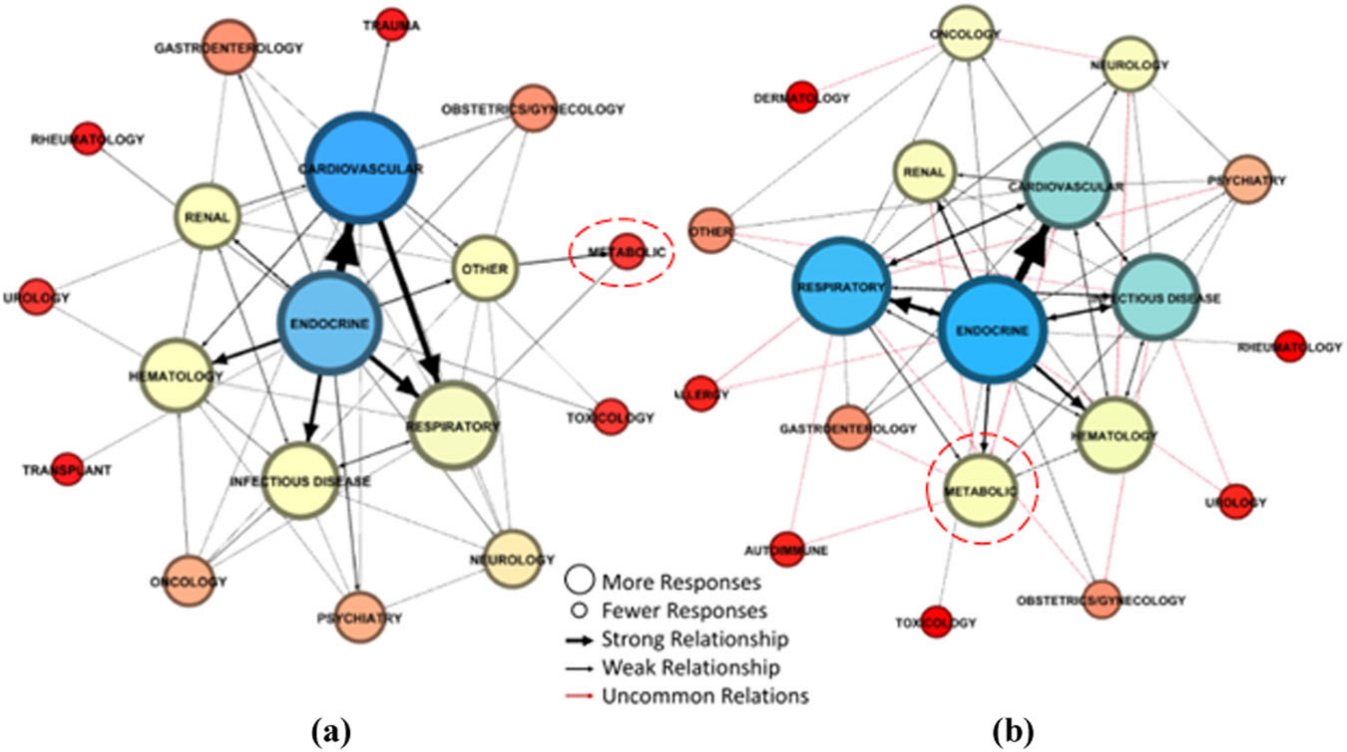
Network analysis of healthcare professional perceptions of the conditions for which POCTs could help them monitor or manage a disease. **a** Before COVID-19, healthcare professionals identified cardiovascular as the condition for which POCTs was most beneficial for monitoring or managing diseases. **b** During COVID-19, healthcare professionals were more focused on using POCTs to monitor or manage endocrine diseases.

Figure 3 presents the network analysis of healthcare professional perceptions of the conditions for which POCTs could help them monitor or manage a disease. Figure 3, panel a illustrates the network analysis of the T1 survey (429 reported conditions). Prior to COVID-19 being designated as a pandemic, healthcare professionals identified 18 medical condition categories for which POCTs could be used to monitor or manage a disease. Cardiovascular and endocrine-related medical conditions featured prominently in the center of the network; suggesting that healthcare professionals considered these conditions to be the most important for which POCTs could be beneficial for monitoring or managing a disease pre-pandemic. The strongest relationships were observed between endocrine → cardiovascular, endocrine → respiratory, and cardiovascular → respiratory.

Figure 3, panel b illustrates the network analysis of the T2 survey (877 reported conditions). Following COVID-19’s designation as a pandemic, healthcare professionals identified 19 medical condition categories for which POCTs could be used to monitor or manage a disease. Endocrine-related medical conditions featured prominently in the center of the network, followed by respiratory, cardiovascular, and infectious disease conditions; suggesting that healthcare professionals considered these conditions to be the most important for which POCTs could be beneficial for monitoring or managing a disease during the pandemic. The strongest relationships were observed between endocrine → cardiovascular and endocrine → respiratory. The red connections (or “edges”) indicate relationships between conditions during COVID-19 that are not common with those pre-COVID-19.

**Fig. 4:**
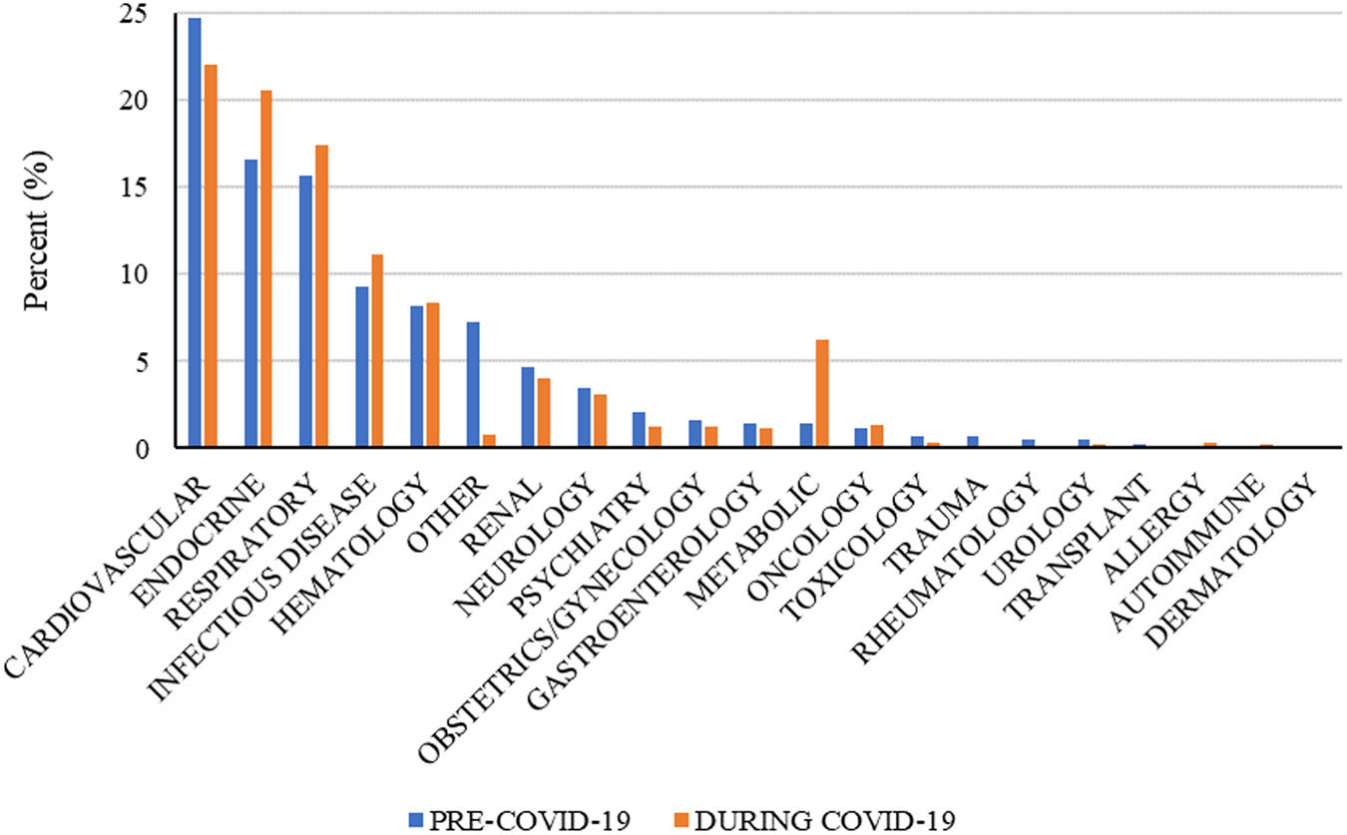
During COVID-19, the metabolic and endocrine categories experienced the greatest percentage increase in healthcare professional attitudes towards using POCTs to monitor or manage diseases.

In Fig. 4, following COVID-19’s designation as a pandemic, there was a 4.9% increase in the metabolic category (*t* = –3.940, *p* < 0.01) and a 4% increase in the endocrine category (*t* = –1.713, *p* < 0.10). Thus, there was a substantial increase in healthcare professional attitudes towards the use of POCTs to monitor or manage diseases in these two categories during the pandemic.

**Fig. 5:**
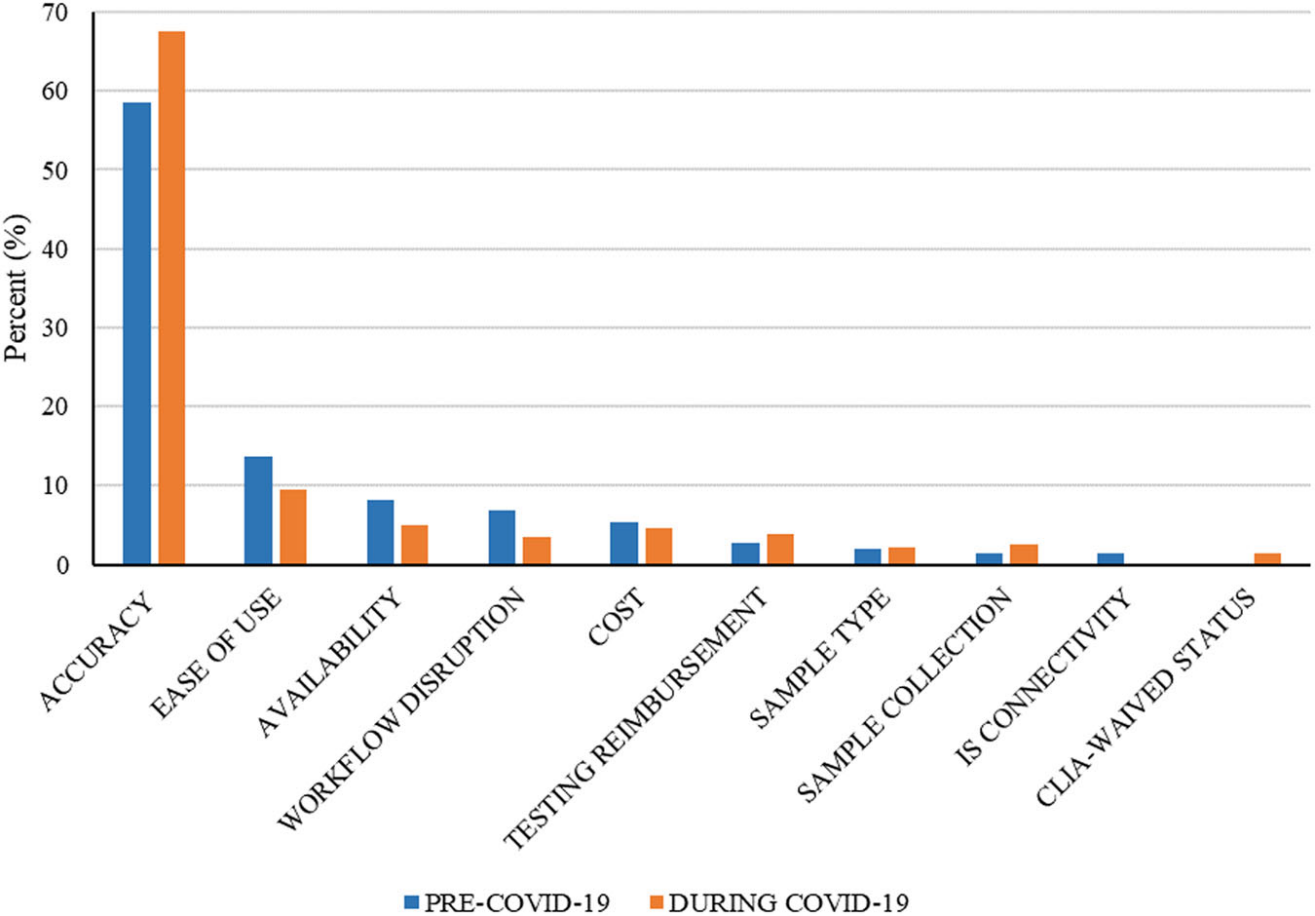
During COVID-19, the accuracy and CLIA-waived status categories experienced the greatest percentage increase among characteristics that healthcare professionals considered important for POCTs.

Figure 5 shows that healthcare professionals considered accuracy to be the most important characteristic of POCTs, followed by ease of use. Following COVID-19’s designation as a pandemic, the POCT characteristics that experienced the greatest increase in healthcare professional perceptions of importance were CLIA-waived status (+100%), sample collection (+44.9%), and testing reimbursement (+30.1%) categories. CLIA stands for the Clinical Laboratory Improvement Amendments, which are a set of regulations enacted in 1988 that apply to all U.S. facilities that “perform laboratory testing on human specimens for health assessment or the diagnosis, prevention, or treatment of disease.”^6^ Under the CLIA regulations, waived tests are relatively simple tests that are cleared for home use by the FDA due to a low risk of erroneous results. CLIA waivers are appropriate for COVID-19 point-of-care tests.^7^

## Discussion

### Perceptions of POCT usage to diagnosis a disease

The results of the network analysis provide important insights into the growing adoption of POCTs to diagnose diseases. Before COVID-19, healthcare professionals identified endocrinology as the category for which point-of-care testing was most helpful for making a medical diagnosis. For example, POCTs in this category may measure blood glucose to help inform healthcare professionals regarding the diagnosis of hypo- or hyperglycemia or may measure hormone levels to help diagnose pregnancy. However, after COVID-19 was designated a pandemic, there was a shift in clinician views of the role of POCTs. Aligned with our expectations, healthcare professionals identified infectious diseases, and COVID-19 in particular, as the category for which POCTs would be most helpful for making a diagnosis, representing an increase of 7.4% in responses. This category migrated to the center of the network.

The cardiovascular category also shifted towards the center of the network. The COVID-19 pandemic was accompanied by an increase in out-of-hospital cardiac arrests (OHCA) among adults (age > 18 years) (Marijon et al., 2020; McVaney et al., 2021). These OHCA cases tended to be localized in areas where COVID-19 was more prevalent (Marijon et al., 2020; McVaney et al., 2021). Prior studies also revealed that hospital visits for acute cardiovascular conditions declined substantially during the pandemic associated with increased fears of being exposed to COVID-19 (Wadhera et al., 2021). This may have contributed to the shift in healthcare professional perceptions regarding a larger role for diagnosing cardiovascular disease. Recent studies have explored the expanding role of POCTs for cardiovascular conditions (e.g., Dunlap et al., 2021).

For a variety of other diseases for which POCTs may not have been much considered, there has been substantial growth. In particular, the metabolic category saw a 4.4% increase following COVID-19’s designation as a pandemic. This increase in healthcare professional perceptions of POCTs being useful for diagnosing disease may be due to a better understanding of metabolic diseases as an important risk factor for severe COVID-19 and increased mortality (le Roux, 2021; Simonnet et al., 2020) or may be related to efficiency of care delivery issues. Furthermore, additional categories including dermatology, autoimmune, and allergy emerged on the periphery of the network during COVID-19. This suggests that the healthcare professional community is thinking much more broadly about POCTs than before the pandemic.

### Perceptions of POCT usage to manage or monitor a disease

The results of the network analyses also provide important insights into the adoption of POCTs to manage or monitor diseases and responses to treatment. Before COVID-19, healthcare professionals identified cardiovascular disease as the category for which point-of-care testing was most helpful for managing or monitoring a disease. However, after COVID-19 was designated a pandemic, there was a shift. Surprisingly, healthcare professionals identified endocrine as the category for which POCTs would be most helpful for monitoring or managing a disease during COVID-19, representing an overall increase of 4%. However, it was the metabolic category that experienced the greatest increase at 4.9%.

Together, endocrine and metabolic diseases have been shown to be important risk factors for severe COVID-19 and increased mortality (le Roux, 2021; Puig-Domingo et al., 2021; Simonnet et al., 2020). The potential for long-term endocrine-metabolic complications that arise from Covid-19 infection (Bornstein et al., 2021; Mongioì et al., 2020) may have raised concerns among healthcare professionals, which led to an increased interest in the adoption of POCTs to manage or monitor endocrine and metabolic conditions. Furthermore, fear of contracting COVID-19 may have kept patients from going to the hospital or clinic, which may have also influenced healthcare professional perceptions about the adoption of POCTs for managing or monitoring these conditions. It is also possible that clinicians experience with POCTs, as an adjunct to successful telemedicine-based encounters for patients with diabetes, has led to higher levels of acceptance.

### POCTs and humanizing healthcare

As our results suggest, the COVID-19 pandemic, being a gray swan event, contributed to a shift in healthcare professional attitudes towards POCTs. In particular, this study reveals that, as a result of shifting perceptions about POCTs, healthcare professionals are becoming increasingly open to the idea of shifting from provider-initiated patient care to empowering patients to become active participants and advocates of their own healthcare via the adoption of novel POCT technologies. This shift from provider-initiated patient care to technology-initiated patient care finds additional support in recent research (e.g., Ding et al., 2020; Teebagy et al., 2022).

The shift in healthcare professional attitudes towards POCTs facilitates the humanization of epidemiology by improving disease management/monitoring. Epidemiologists have long recognized the value of POCTs for tracking the spread of infectious diseases (Informa, 2020). While the global demand for COVID-19 diagnostic tests is behind the recent expansion of the POCT market, the pandemic has also fueled significant growth in other segments as well, such as oncology, blood screening, and genetic disorders (Informa, 2020). Advances in diagnostic technologies that enable rapid turnaround times, increase test sensitivity, reduce invasiveness, and lower costs contributed to this growth (Informa, 2020). These advances provide healthcare professionals with more options from which they may draw from in order to manage and monitor patients afflicted with various diseases.

To illustrate how the use of POCTs impacted patient care at their practice, we draw upon interviews of healthcare professionals. In the context of the pandemic, healthcare professionals were on the front lines and found that the use of rapid COVID-19 tests provided them with peace of mind because they had foreknowledge of whether the patient about to be seen was infected with COVID-19. This allowed healthcare professionals to focus on diagnosing and treating the patient. One physician interviewed stated “I know before I step into the examination room whether the patient has COVID, unlike the general population. If they tested positive, I can take extra precautions to protect myself. I feel very safe treating my patients and this allows me to focus on my bedside manner.” Having this information beforehand was viewed as helping to improve the clinician-patient relationship during the pandemic. While there were some concerns among physicians about POCT technologies making the diagnosis in lieu of the physician, there was a general consensus that POCTs helped improve patient outcomes because it facilitated dialog that helped build the clinician-patient relationship, thereby bringing the humanity back into the examination room quicker than during other epidemics (e.g., HIV).

From the patient perspective, the adoption of novel POCTs allows them to become better-informed consumers of healthcare by obtaining their own healthcare data in real-time. In this manner, patients can seek immediate help from healthcare professionals for conditions that are time sensitive (e.g., cardiovascular disease) or that require patient engagement to achieve outcomes that improve the quality of life (e.g., diabetes). Furthermore, the variation in specific health outcomes can be reduced by timely and accurate point-of-care testing that engages patients in managing their condition(s). In practice, there are few problematic health conditions that are cured without patient management. Indeed, patient-generated POCT healthcare data can be shared with physicians to facilitate a more accurate diagnosis of a health condition because it can be obtained at the time when the health event occurred (and not hours or days later when the patient can secure an appointment with a clinician). Thus, the data that patients obtain from POCTs can help further the humanization of epidemiology by facilitating greater dialog between clinicians and patients, thereby strengthening this important relationship.

### Limitations and future research

We would caution against the generalization of our results beyond the U.S. context. Since the majority of our survey respondents were from the U.S., the views in our study largely reflect those of U.S. healthcare professionals. Although we received a few responses to our surveys from healthcare professionals located outside the U.S., our intent was not necessarily to examine healthcare professional perceptions of POCTs on a global context. Further, since the U.S. is the largest market for POCTs (Informa, 2020), this may also have influenced healthcare professional attitudes as these diagnostic tests tend to be more readily available in the U.S. market. Future studies may explore perceptions of the use of POCTs among healthcare professionals from other countries, especially in less developed countries where access to high-quality healthcare may be limited or where insurance reimbursements for POCTs may not be available. Another limitation is that, due to the anonymity of our survey respondents, we are unable to match pre-COVID and during COVID responses among individuals. Thus, we are only able to measure changes in healthcare professional attitudes towards POCTs in aggregate. Future studies may delve into the drivers behind the shift in individual healthcare professional attitudes towards POCTs (e.g., education, prior experience, unmet need, insurance reimbursement, etc.). We are also limited in our ability to attribute causality by the observational nature of this study. Future studies may attempt to empirically model and test the shift in healthcare professional attitudes towards the adoption of POCTs using, for example, a difference-in-difference or propensity score matching statistical analysis technique in which causality can be more easily attributed.

## Conclusion

It is not uncommon for healthcare professionals to be intimately involved in the development and adoption of innovative medical solutions. Indeed, healthcare professional perceptions of POCTs are increasingly relevant and guide research solicited through government programs, such as the Point of Care Technology Research Network (POCTRN).^8^ In this regard, the shift in healthcare provider attitudes towards POCTs is an important factor that contributes to accelerate the development of these nascent technologies and spurs their continued adoption. Thus, healthcare professionals of all disciplines are encouraged to engage in these collaborative research initiatives to provide a critical voice that shapes the future development of POCTs as this may better prepare us for the next gray swan event.

While the adoption of COVID-19-related POCTs was driven by an overwhelming need to diagnose whether patients were infected with the virus, it had the unintended consequence of bringing to light areas where healthcare professionals could apply POCTs more broadly to help diagnose, manage, and monitor various diseases, including cardiovascular, endocrine, respiratory, metabolic, and other infectious diseases. The increased recognition of the utility of POCTs among healthcare professionals, along with enhancements in accuracy and ease of use, suggests that they can be considered a valuable tool to help diagnose, manage, and monitor diseases. While POCTs will not obsolete traditional lab tests, they represent an opportunity for patients afflicted with diseases to become empowered and more engaged in improving their overall health and quality of life. Thus, as a tool for both healthcare professionals and patients, POCTs can help humanize epidemiology by facilitating constructive dialog that elevates patient care. Healthcare professionals are, therefore, encouraged to collaborate with patients by reviewing their POCT data and assessing how it might fit and inform the diagnosis, management, and monitoring of diseases. In short, by working with healthcare professionals, patients should embrace the use of POCTs as these tools can be integrated into a patient’s overall strategic healthcare plan.

It should be noted that infectious disease research had been on the decline for many years prior to the COVID-19 outbreak (Lloyd, 2020). As a result, governments and healthcare systems, around the world, were caught largely unprepared, and many assumptions about our understanding of infectious diseases were challenged. However, the differences in clinician views that we observed towards POCTs may drive technological advancements in this domain that may better prepare us for the next gray swan event. Since healthcare professionals are instrumental in the development of innovative medical technologies, we can prepare for the next unexpected healthcare event by investing in the development of biomedical and genomic technologies, such as new polymerase chain reaction (PCR) and antigen capture technologies that are ultra-sensitive, low cost, and rapidly programmable that can be developed into POCTs (Botti-Lodovico and Sabeti, 2022).

## Data Availability

The datasets generated during and/or analyzed during the current study are available in the UMass Chan repository, Data File #1004, 10.13028/dtb5-q194.

## Footnotes

1 Source: https://reporter.nih.gov/.

2 Source: https://profiles.umassmed.edu/search/.

3 Source: http://direct2experts.org/.

4 Only one response from the T1 survey was collected on March 25, 2020, which came after March 11, 2020—the date when the World Health Organization (WHO) declared COVID-19 a global pandemic. Thus, the T1 survey can be largely considered to measure pre-pandemic attitudes of POCT usage among healthcare professionals while the T2 survey measures post-pandemic attitudes of POCT usage among healthcare professionals.

5 Since diabetes can be considered both an endocrine and metabolic condition, for the purpose of this study, we categorized diabetes as an endocrine condition.

6 Source: https://www.cdc.gov/labquality/waived-tests.html.

7 Source: https://www.cdc.gov/coronavirus/2019-ncov/lab/point-of-care-testing.html.

8 The Point of Care Technology Research Network is a network of universities (i.e., UMass Chan Medical School/UMass Lowell, Johns Hopkins, Northwestern, and Georgia Tech) and industry partners that develops technologies with clinical applications that enhances complementary strengths and builds multidisciplinary partnerships. POCTRN was instrumental in helping with the RADx effort to bring new POCTs to market.
